# Exploring the Potential Role of Metabolomics in COPD: A Concise Review

**DOI:** 10.3390/cells13060475

**Published:** 2024-03-07

**Authors:** Claudio Tirelli, Sabrina Mira, Luca Alessandro Belmonte, Federica De Filippi, Mauro De Grassi, Marta Italia, Sara Maggioni, Gabriele Guido, Michele Mondoni, Giorgio Walter Canonica, Stefano Centanni

**Affiliations:** 1 Respiratory Unit, ASST Santi Paolo e Carlo, Department of Health Sciences, University of Milan, 20142 Milan, Italy; 2 Personalized Medicine, Asthma and Allergy, IRCCS Humanitas Clinical and Research Center, 20089 Rozzano, Italy; 3 Department of Biomedical Sciences, Humanitas University, Pieve Emanuele, 20072 Milan, Italy

**Keywords:** Chronic Obstructive Pulmonary Disease, metabolomics, mass spectrometry, nuclear magnetic resonance, exhaled breath condensate

## Abstract

Chronic Obstructive Pulmonary Disease (COPD) is a pathological condition of the respiratory system characterized by chronic airflow obstruction, associated with changes in the lung parenchyma (pulmonary emphysema), bronchi (chronic bronchitis) and bronchioles (small airways disease). In the last years, the importance of phenotyping and endotyping COPD patients has strongly emerged. Metabolomics refers to the study of metabolites (both intermediate or final products) and their biological processes in biomatrices. The application of metabolomics to respiratory diseases and, particularly, to COPD started more than one decade ago and since then the number of scientific publications on the topic has constantly grown. In respiratory diseases, metabolomic studies have focused on the detection of metabolites derived from biomatrices such as exhaled breath condensate, bronchoalveolar lavage, and also plasma, serum and urine. Mass Spectrometry and Nuclear Magnetic Resonance Spectroscopy are powerful tools in the precise identification of potentially prognostic and treatment response biomarkers. The aim of this article was to comprehensively review the relevant literature regarding the applications of metabolomics in COPD, clarifying the potential clinical utility of the metabolomic profile from several biologic matrices in detecting biomarkers of disease and prognosis for COPD. Meanwhile, a complete description of the technological instruments and techniques currently adopted in the metabolomics research will be described.

## 1. Introduction

Chronic Obstructive Pulmonary Disease (COPD) is a pathological condition of the respiratory system characterized by chronic airflow obstruction, associated with changes in the lung parenchyma (pulmonary emphysema), bronchi (chronic bronchitis), and bronchioles (small airways disease). The disease presents a non-reversible obstruction in pulmonary function tests. Environmental factors, exposure to toxic inhalants, along with genetic and epigenetic alterations, can contribute to the pathogenesis of the disease [1,2]. Oxidative stress and changes in lipid and amino acid metabolism contribute to the development of the disease. COPD is clinically characterized by symptoms such as chronic cough, dyspnea, increased bronchial secretions, and impaired exercise tolerance. COPD is associated with poor outcomes and chronic disability. In Italy, the disease affects 5.6% of adults (approximately 3.5 million people) and is responsible for 55% of deaths from respiratory diseases [3]. Since COPD is a complex and multifaceted disease, the concepts of phenotypes (i.e., subgroups with prognostic impact) and endotypes (i.e., conditions defined by specific pathophysiological and molecular mechanisms) have begun to be applied to achieve the goal of personalized medicine and a more efficient diagnostic and therapeutic approach.

Metabolomics is the science that investigates the metabolites present in a biological sample, thus inferring the metabolic and biological pathways behind their production.

Metabolomics specifically refers to the systematic analysis and determination of specific analytes (metabolites) present in a biological matrix, aiming to identify a set of biomarkers for diseases or metabolic processes [4].

Metabolomics thus seems to be a useful scientific tool to enhance our understanding of disease mechanisms and to complement what genetics can already explain about lung diseases. In the future, it may not only aid in obstructive diseases but also in restrictive diseases, such as Interstitial Lung Diseases (ILD) [5,6].

Metabolites are small biomolecules (<1500 Daltons) that represent the phenotypic product of specific gene expression. Metabolites can include amino acids, organic acids, nucleic acids, peptides, carbohydrates, and inorganic molecules, which can be found in both cells and bio-fluids [7]. These small molecules are involved in biological processes, such as bio-signaling, apoptosis, and oxidative stress [8].

COPD pathophysiology involves several metabolic processes that can be studied through the analysis of their metabolites using metabolomic techniques. These analyses can help identify metabolomics profiles that characterize COPD patients in terms of disease severity, exacerbation rate, and level of obstruction [9].

**Figure 1:**
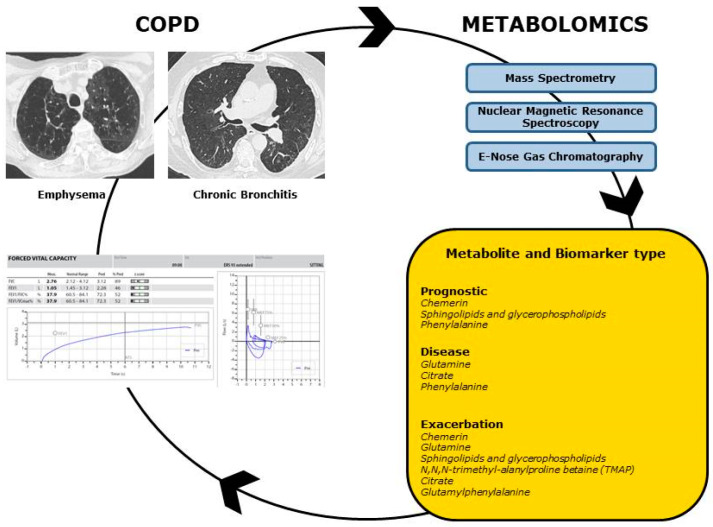
Metabolomics in COPD. Metabolomic profiles could be useful to better characterize COPD phenotypes and endotypes, with correlation to functional and radiologic data. Through the adoption of the main Metabolomic techniques (Mass Spectrometry, Nuclear Magnetic Resonance Spectroscopy, E-Nose Gas Chromatography), metabolites can be identified as prognostic, disease and exacerbation biomarkers.

Today, the determination of metabolomic profiles can be obtained through various analytical methods and technological instruments, primarily based on mass spectrometry (MS) and nuclear magnetic resonance spectroscopy (NMR). Exhaled breath condensate (EBC), plasma, and bronchoalveolar lavage fluid (BALF) are among the most commonly used biomatrices for metabolomics analysis [10]. The aim of this review was to comprehensively analyze the relevant literature on the applications of metabolomics in COPD, elucidating the potential clinical utility of metabolomic profiles from various biological matrices in detecting biomarkers for disease and prognosis in COPD. Meanwhile, a complete description of the technological instruments and techniques currently adopted in the metabolomics research will be described (Figure 1).

## 2. Materials and Methods

A non-systematic narrative literature review was conducted. The PubMed and Scopus search engines were used to collect the relevant articles from the international literature until November 2023. The search strategy was conducted by adopting combinations of the following keywords and including articles written in English only: COPD, metabolomics, metabonomics, biomarker, mass spectrometry, nuclear magnetic resonance spectroscopy, E-nose gas chromatography, exhaled breath condensate, diagnosis, accuracy, monitoring, exacerbation, screening. Additional eligible studies were retrieved from bibliographies of most cited studies and book chapters. Two authors (C.T. and S.Mi.) independently screened abstracts from all the collected articles; in case of contrasting opinion, a third author (M.M.) screened and decided whether to include the paper for analysis. Full texts of the included articles were examined and relevant data summarized.

## 3. Analytical Techniques in Metabolomics

Metabolomic profiles can be obtained through the application of various analytical techniques. A comprehensive identification and quantification of a metabolomic profile relies on multiple analytical platforms. Mass Spectrometry (MS) and Nuclear Magnetic Resonance Spectroscopy (NMRS) are the most commonly used metabolomics methods. While mass spectrometry (MS) specifically identifies metabolites once they have been separated (i.e., by gas or liquid chromatography and capillary electrophoresis), nuclear magnetic resonance spectroscopy (NMRS) allows for the identification of metabolites without a preemptive separation phase (Table 1) [11].

Even though NMRS and MS are currently regarded as the preferred methods for metabolomics investigations, no single analytical platform can comprehensively identify and quantify the metabolome of a biological system due to its intrinsic chemical diversity. Therefore, a combination of various analytical techniques should be favored as it can yield complementary results [12]. More recently, E-nose chromatography has been studied as an innovative methodology for analyzing volatile organic compounds in metabolomics studies [13].

### 3.1. Mass Spectrometry

Mass spectrometry (MS) is a technique that allows for the determination of the masses of molecules present in a biological sample. Mass spectrometry (MS) ionizes molecules and categorizes ions based on their mass-to-charge ratio (*m*/*z*). Mass spectrometry methods vary in terms of analysis time, sensitivity, selectivity, robustness, ease of use, and cost.

In mass spectrometry, ions are generated and separated from molecules in an ion source using a mass analyzer. Mass analyzers can exist in various formats with increasing mass resolution and accuracy, such as quadrupole, ion trap, time-of-flight, Orbitrap, and Fourier Transform Ion Cyclotron Resonance [12,14].

More specifically, once generated from molecules, ions are separated based on the mass-to-charge ratio (*m*/*z*) in the mass analyzer. Electric charge propels ions through the high vacuum space of the spectrometer, influenced by static or dynamic electric or magnetic fields, or even field-free regions, depending on the type of analyzer. The resulting mass spectrum displays detected ions with their relative abundance, providing information about molecular weight [15]. Compared to NMRS, MS offers higher intrinsic sensitivity and specificity, although it typically requires a preceding separation step, such as gas chromatography (GC), high-performance liquid chromatography (HPLC), ultra-performance liquid chromatography (UPLC), or capillary electrophoresis (CE) [15]. The integration of separation techniques with mass spectrometry is essential for decreasing sample complexity and minimizing ionization suppression effects. This enhances detection sensitivity and broadens metabolome coverage. MS sample preparation involves techniques such as lyophilization, liquid–liquid extraction (LLE), solid-phase extraction (SPE), and micro-extraction (SPME) [12].

MS-based metabolomics allows for both untargeted and targeted approaches. The untargeted strategy assesses the entire molecular content of samples, facilitating a more comprehensive understanding of physiological and pathological biochemical processes. Conversely, targeted methodology reveals the biological mechanism(s) underlying pathology. It identifies and quantifies specific metabolites or classes of metabolites based on a hypothesis-driven strategy but overlooks potential new or unknown disease biomarkers [10,12].

The application of gas chromatography-mass spectrometry (GC-MS) and liquid chromatography-tandem mass spectrometry (LC-MS/MS) for analyzing volatile and semi-volatile, thermally stable compounds is particularly valuable, especially in exhaled breath condensate (EBC). GC-MS is effective in detecting such compounds, and with appropriate chemical derivatization, non-volatile compounds can be transformed into volatile forms for analysis. The identification of volatile organic compounds (VOCs) in exhaled breath condensate (EBC) is typically conducted using gas chromatography–mass spectrometry (GC-MS). Unidentified mass values necessitate mass spectral databases for matching molecular ion and ion fragment patterns. Reference standards are recommended for structural validation due to the incomplete coverage of databases [10,12].

Indeed, human breath contains hundreds of volatile organic compounds (VOCs), including inorganic molecules (e.g., NO, O_2_, CO_2_), organic compounds such as hydrocarbons, terpenes, alcohols, aldehydes, and ketones, as well as sulfur and nitrogen compounds detectable by GC-MS. Recent investigations also include more polar, less volatile, or non-volatile organic compounds extracted through liquid–liquid extraction (LLE) or solid-phase extraction (SPE) methods. Eicosanoids, such as leukotrienes, isoprostanes, prostaglandins, and thromboxanes, are considered candidate biomarkers for inflammation and oxidative stress. They require GC-MS analysis after suitable derivatization due to their chemical features [16].

LC-MS/MS utilizes multiple platforms for the simultaneous identification and quantification of targeted metabolites, such as 8-isoprostaglandin F2α, in human exhaled breath condensate (EBC) samples. This method provides high sensitivity and specificity, frequently eliminating the need for sample derivatization. It is particularly suitable for investigating thermally unstable molecules that are not safe for GC-MS-based methodologies [17].

LC-MS/MS, particularly in tandem MS mode, has been widely employed for the precise measurement of targeted classes of metabolites. These include markers of inflammation, such as airway aldehydes, eicosanoids, phospholipids, lysolipids, glucose, amino acids, as well as their catabolites and purines. In evidence-based care (EBC), liquid chromatography–tandem mass spectrometry (LC-MS/MS) is primarily used to assess inflammation biomarkers in individuals with various health conditions. Furthermore, LC-MS/MS proves valuable for the analysis of proteins in Exhaled Breath Condensate (EBC) samples, as demonstrated in lung cancer screening and asthma monitoring [10].

The advent of mass spectrometry (MS), including gas chromatography–mass spectrometry (GC-MS) and liquid chromatography–mass spectrometry (LC-MS), has made proteomics and metabolomics more feasible in large-scale population studies [18]. All these technologies have the potential to process hundreds of samples per day, with the limitation in the case of mass spectrometry (MS) being determined by the chromatographic run times. GC-MS and LC-MS methods typically exhibit higher sensitivity than NMR spectroscopy—sometimes by several orders of magnitude. However, in the case of LC-MS, quantification relies on ionization efficiency and peak overlap. Despite these considerations, both GC-MS and LC-MS demonstrate high reproducibility. Moreover, these technologies are cost-effective when compared to other omics approaches such as proteomics and gene arrays. Nevertheless, achieving absolute quantification with LC-MS can be challenging unless a standard is available for comparison [14].

### 3.2. Nuclear Magnetic Resonance Spectroscopy

Nuclear Magnetic Resonance Spectroscopy (NMRS) is a non-destructive, non-invasive technique for detecting metabolites. It has the advantage of requiring minimal sample pretreatment, providing a rapid metabolic profile of the sample, and allowing for multiple sample analyses [19].

In nuclear magnetic resonance spectroscopy (NMRS), samples are investigated at the atomic scale by placing them in a static magnetic field and exposing them to electromagnetic radiation with a specific resonance frequency. This frequency depends on the strength of the magnetic field and the magnetic properties of the nucleus. The electromagnetic wave emitted when the nuclei return to the fundamental (“relax”) state is thus recorded. The “spectrum” is the result of the Fourier transformation of the recorded signal.

Biological molecules are mainly composed of NMR-detectable isotopes (hydrogen, carbon, oxygen, nitrogen, and fluorine atoms), and the amplitude of the response linearly depends on sample concentration. The higher the static magnetic field, the more spectral details are detected. The highest magnetic field strength reached so far is 28.2 T (1.2 GHz for proton frequency), but most metabolomic applications use spectrometers operating at 14.1 T (600 GHz), equipped with CryoProbe technology, which increases sensitivity and reduces electronic thermal noise [20].

Settings for acquisition (excitation pulse width, receiver gain, acquisition intervals, relaxation times, and solvent suppression parameters) and processing (apodization, phasing, baseline correction, selection of integral regions, peak alignment, normalization, centering, scaling, and transformation) need strict control and must be carefully selected by researchers [21].

Metabolomics analysis should aim to reduce the large amount of data collected by identifying redundant or unnecessary variance and deriving statistically significant information. Dedicated software or free online platforms can generate projections of the acquired dataset in their respective latent spaces, enabling intuitive data visualization and dimensionality reduction. Metabolomic analyses finally help identify a specific pathophysiological state by characterizing the fundamental metabolic pathways and classifying specific phenotypes/endotypes. Possible biomarkers of disease can be obtained by comparing metabolomic profiles derived from patients (i.e., COPD) with those derived from healthy controls.

### 3.3. E-Nose Gas Chromatography

E-nose technology is an innovative methodology for analyzing exhaled breath and detecting volatile organic compounds (VOCs). It utilizes an array of sensors functionalized with chemical or biological materials to interact with volatile organic compounds (VOCs) in exhaled air, including options such as metal oxides, organic polymers, nanotubes, and nanowires. Studies are focusing on the potential application of E-nose in the diagnosis and monitoring of respiratory diseases [22].

E-nose employs two interpretative approaches: direct and indirect. Direct analysis visualizes volatile organic compound (VOC) patterns through graphical or numerical representations, such as radar plots, histograms, and score plots. In contrast, indirect interpretation explores correlations between volatile organic compound (VOC) patterns and known indicators of specific conditions or diseases. Studies on E-nose applications in respiratory diseases, such as COPD, asthma, and lung cancer, indicate its ability to discriminate between patients and healthy controls. Moreover, considering the extreme simplicity of modern methods for collecting exhaled breath, volatile organic compounds (VOCs) could be especially beneficial for the increasing population of patients (elderly, children, and noncompliant subjects) who may struggle to undergo standard diagnostic procedures. However, further evidence and standardization are required before clinical integration [23].

Despite its potential, E-nose technology faces significant challenges, including the lack of a critical mass of observations, model heterogeneity, and the need for long-term studies. Additional issues involve the absence of standardization in sample collection and the necessity for physicians to interpret complex data.

Comparing electronic nose (E-nose) technology with mass spectrometry (MS), it becomes evident that E-nose offers a swift and non-invasive method for analyzing volatile organic compounds (VOCs) but encounters challenges related to insufficient critical observations and model heterogeneity. Conversely, mass spectrometry (MS), a more established technique that uses ion separation to identify and quantify compounds, is renowned for its precision. Although mass spectrometry can be costly and requires intricate instruments, it excels in scientific precision. The synergy between these technologies has the potential to advance towards a more comprehensive and personalized diagnosis of respiratory conditions.

Despite its limitations, the analysis of volatile organic compounds (VOCs) in exhaled breath shows innovative potential for diagnosing and managing lung diseases. Significant and collaborative efforts are needed to overcome technical and methodological challenges, paving the way for E-nose technology to become an accepted diagnostic procedure in everyday clinical practice through the ongoing integration of clinical and technological research [24].

## 4. Biological Matrices for Metabolomic Studies in COPD

Phenotyping COPD is an essential goal for clinicians. Metabolomics can be a valuable tool for identifying and quantifying the small molecules produced during biological processes [25]. Furthermore, metabolomics might help in evaluating which metabolic pathways are altered by COPD pathophysiology, thus assisting in identifying possible novel diagnostics and treatment response biomarkers [26]. A growing number of studies have been dedicated to the identification of metabolites in biological matrices from COPD patients. The metabolomics characterization was conducted on a diverse set of biological samples, including exhaled breath condensate (EBC), serum, plasma, bronchoalveolar lavage fluid (BALF), sputum, urine, and stools. A detailed summary of the principal metabolites based on the specific biological matrix is provided in Table 2.

### 4.1. Exhaled Breath Condensate

EBC is a natural matrix that can be easily, non-invasively, and repeatedly obtained by directing a subject’s breath (from spontaneous tidal breathing or from patients undergoing mechanical ventilation) over a cold surface. Exhaled breath condensate (EBC) contains mostly water (99.9%), but inorganic compounds are often present, such as nitric oxide and carbon monoxide, volatile organic compounds (VOCs), as well as non-volatile substances. These components reflect the composition of the airway-lining fluid (ALF). The non-volatile compounds primarily consist of inorganic anions and cations, proteins, surfactants, and other organic molecules (such as amino acids, organic acids, and urea).

EBC analysis is an effective method for sampling the airway-lining fluid (ALF), which reflects airway inflammation and mirrors the severity of lung injury [16]. The nature and source of the exhaled particles in exhaled breath condensate (EBC) are still debated, as well as the mechanism by which these particles form and change during exhalation. While the presence of volatile organic compounds (VOCs) can be explained by the partitioning between the gaseous and aqueous phases of the exhaled breath, the exact mechanism clarifying how the non-volatiles enter the gaseous phase remains unknown. One of the most accepted theories is that turbulent airflow during exhalation releases small amounts of ALF from the airway lumen surface [27]. Many external factors influence EBC composition, such as sex, age, and infections. Moreover, since exhaled breath condensate (EBC) is mainly composed of water, the compounds in EBC are highly diluted, with a dilution factor ranging between 1000 and 50,000 [28]. The appropriate dilution standardization must always be addressed for the correct application of metabolomic-based analysis of exhaled breath condensate (EBC) in clinical and research settings. Several studies have recently proposed using NMR-based metabolomics of exhaled breath condensate (EBC) as a rapid non-invasive tool for investigating airway diseases.

### 4.2. Plasma and Serum

Different studies have focused on identifying the metabolites in the plasma and serum of COPD patients.

Blood plasma and serum (which differ due to the presence of clotting compounds) are water-based solutions rich in carbohydrates, lipids, amino acids, proteins, peptides, and electrolytes [26]. Plasma and serum metabolite composition can be altered by various stimuli and toxins (e.g., cigarette smoke).

Serum metabolomics proved effective in distinguishing healthy subjects from individuals with asthma and COPD. Particularly, asthma and COPD appear to differ in terms of 16 specific metabolites [29,30]. Analysis of peripheral blood helped identify a specific metabolomic profile in COPD with emphysema compared to chronic bronchitis with high sensitivity and specificity. Emphysema patients showed decreased levels of serum creatine, glycine, and N,N-dimethylglycine [28]. Moreover, blood plasma/serum biomarkers (e.g., white blood cell (WBC) counts, IL-6, CRP, IL-8, fibrinogen) have been utilized in predictive models for assessing the risk of death in COPD [31,32].

### 4.3. Bronchoalveolar Lavage Fluid

BALF collected from COPD patients represents a biomatrix rich in metabolites that can be studied for metabolomic profiles [33,34].

Halper-Stromberg et al. analyzed metabolites of bronchoalveolar lavage (BAL) in COPD patients based on phenotypes: bronchial obstruction and emphysema. In COPD patients with a reduced FEV1/FVC ratio, the bronchoalveolar lavage (BAL) is enriched in amino acids (arginine, isoleucine, and serine), fatty acids, and various phospholipids such as phosphatidylethanolamines, lysophosphatidylethanolamines, lysophosphatidylcholines, phosphatidylserines, phosphatidylinositols, and phosphatidylcholines. BAL analysis of patients with emphysema revealed a significant increase in the same metabolites, except for lysophospholipids and phosphatidylserines, and an additional presence of carnitine [35].

In a study on bronchoalveolar lavage (BAL) conducted on healthy individuals, non-smokers, smokers, and subjects with COPD, the main difference found was between female smokers with normal lung function and female patients with COPD. The different metabolites in BAL include nine lipid mediators, such as epoxy-octadecenoic acids and dihydroxy-octadecenoic acids. These substances increase the permeability of intercellular junctions, are correlated with the abundance of goblet cells, which increases with smoking, and can influence the activity of cytochrome P450 [36].

During exacerbation, an increased expression of heparan sulfate and chondroitin sulfate can be observed in bronchoalveolar lavage (BAL). These are related to the function of matrix metalloproteinases (MMP-2, MMP-9, and MMP-12), which are associated with airway tissue remodeling during the acute exacerbation stage of COPD [37].

### 4.4. Urine

Urine is a water based biological matrix, which can be analyzed to detect metabolites. Particularly, different studied have tried to compare metabolite composition in urine and serum of COPD patients. It has been found that in urine of COPD patients 1-Methylnicotinamide (1-MN), creatinine and lactate were reduced when compared to healthy subjects. Moreover, in urine of COPD patients elevated level of acetate, acetoacetate, acetone, carnosine, hydroxyphenylacetate, phenylacetylglycine, pyruvate and a-ketoglutarate were detected. The increase of acetate, that is the final product of lipid metabolism, in associated with accelerated lipid catabolism, which is present in COPD patient for their poor nutritional status. The production of ketone bodies, acetoacetate and acetone, may indicate the utilization of storage lipids as an alternative energy resource [38].

### 4.5. Induced Sputum

Induced sputum can also be used to study and analyze metabolites in COPD patients. Induced sputum is performed by inhaling a hypertonic saline solution (at concentrations ranging from 3% to 5%) through an ultrasonic nebulizer. A study analyzed induced sputum from 80 patients with COPD and 37 healthy controls to measure lipid mediators and cells that could potentially reflect the inflammatory process in the bronchial tree of COPD subjects. In this study, an increased sputum eosinophilia was found in COPD subjects, which is correlated with blood eosinophilia. Moreover, leukotriene E4, leukotriene D4, prostaglandin D2, prostaglandin E2, 8-iso-prostaglandin E2, 8-iso-prostaglandin F2a, 5-oxo-eicosatetraenoic acid, 12-oxo-eicosatetraenoic acid, and 11-dehydro-thromboxane B2 have a higher concentration in induced sputum in COPD subjects compared to healthy controls. In COPD subjects with disease exacerbations during the past 12 months, there were higher concentrations of prostaglandin D2, 12-oxo-eicosatetraenoic acid, and 5-oxo-eicosatetraenoic acid [39].

Sputum can also be a useful predictor biomarker of exacerbations. In patients with COPD, six metabolites were found to be altered compared to healthy non-smokers: sialic acid, hypoxanthine, xanthine, methylthioadenosine, adenine, and glutathione. Stratifying patients according to the Global Initiative for Chronic Obstructive Lung Disease (GOLD) stages, sialic acid was found to be elevated in all groups, while hypoxanthine and xanthine were elevated in those with more severe disease. Sialic acid can be used as a biomarker for mucin concentration and bronchitis. Both sialic acid and hypoxanthine were found to be more elevated in patients experiencing common pulmonary exacerbations. Therefore, they could potentially serve as biomarkers for predicting future exacerbations and identifying patients at a higher risk for such events [40].

### 4.6. Stools

Stools can be also used in metabolomics studies to identify potential biomarkers. In a study conducted in Australia, metabolic profiles of stools from 28 COPD patients and 29 healthy controls were analysed. Some metabolites (lipid, amino acid and xenobiotic classes) were significantly different between COPD and healthy people after adjustments for covariates, like age, sex or BMI. Particularly, N-acetylcadaverine, N-acetyltaurine and the tobacco metabolite cotinine were detected at higher concentrations in COPD [41,42].

### 4.7. Lung and Bronchial Tissue

Metabolomic studies on lung and bronchial tissue are still limited. The severity of COPD and the presence of emphysema have been linked to phosphatidylinositol and phosphatidylserine profiles in lung tissue [43]. Particularly, higher levels of ceramides have been found in mild and moderate COPD, reflecting the extent of parenchymal involvement and damage. Lower levels of ceramides and higher levels of phospholipid sphingosine-1-phosphate were mostly observed in severe COPD [44].

## 5. Application of Metabolomics in the Study of COPD: Biomarkers of Disease and Prognosis

Metabolomics could provide an opportunity to comprehensively characterize COPD within the realm of obstructive lung diseases, not only in a research environment but also in the identification of biomarkers that could be applied in clinical settings in the future. Indeed, obstructive lung diseases may present with similar symptoms, even if their anatomical and biological bases are profoundly different. Misdiagnosis can lead to less effective treatment and failure to control symptoms. In this sense, several groups have started to identify specific metabolomic profiles to distinguish COPD from other conditions. Through the study of individual metabolic profiles, clinicians can theoretically identify specific metabolic alterations in patients. This allows for the personalization of treatment plans based on the patient’s metabolic phenotype. Metabolomics can help identify potential biomarkers of COPD that coincide with dysregulated metabolites in COPD. These biomarkers can be classified as: (1) Disease biomarkers; (2) Exacerbation biomarkers; (3) Prognostic biomarkers, particularly in terms of disease progression; (4) Response to treatment biomarkers (refer to Table 3).

It has been reported that serum and plasma metabolites of inflammation and oxidative stress could help recognize the development and exacerbation of COPD [40]. Moreover, plasma and sputum sphingolipid levels were higher in emphysema and COPD exacerbations [55].

Maniscalco et al. found that nuclear magnetic resonance spectroscopy (NMRS) analysis of exhaled breath condensate (EBC) from COPD patients showed increased levels of ethanol and methanol and decreased levels of formate and acetone/acetoin when compared to EBC from asthmatic patients [58]. Bertini et al. found that a persistent pro-inflammatory environment in COPD airways could be associated with high lactate levels in exhaled breath condensate (EBC) compared to healthy subjects. Other metabolites that were highly represented include acetate, propionate, serine, proline, and tyrosine [59]. De Laurentiis et al. described a different metabolomic profile in two smoking-related obstructive diseases, namely COPD and pulmonary Langerhans cell histiocytosis (PLCH) [60]. While both of these diseases showed increased acetate levels compared to healthy non-smokers controls, COPD exhibited high levels of 2-propanol and low levels of isobutyrate, whereas PLCH showed exactly the opposite trends. All of this is preliminary data obtained in research settings, with the potential for further investigation towards future clinical applicability. In this way, metabolomics, by identifying alterations in metabolites or metabolic pathways associated with disease, could offer insights into the underlying pathophysiological processes of COPD. This could play an important role in developing targeted therapies.

In more detail, regarding alterations in glucose-lipid metabolism and oxidative stress-related pathways, metabolomics studies have shown that:

- *Phospholipid-derived sphingomyelins and glycerophospholipids* metabolism are altered in COPD, causing endothelial defense mechanism dysregulation, alveolar epithelial cell apoptosis induction, promotion of inflammatory response, and macrophage dysfunction due to ceramide accumulation in lung tissue [61]. This alteration also leads to dysfunction in vascular endothelial cells by activating NF-κB and promoting the inflammatory production of cytokines, mediated by lyso-phospholipids accumulation in lung tissue [62]. Consequently, there is an increase in oxidative stress, phospholipid oxidation, and activation of the innate immune system, resulting in persistent inflammation [63].
- *Chemerin*, an adipokine secreted primarily by white adipose tissue, adiponectin is a regulator of inflammation, glucose, and lipid metabolism. These elements coexist and interact in COPD patients, making adiponectin a potential therapeutic target. Particularly in the context of exercise-mediated lung rehabilitation, its expression is influenced by exercise in vivo [45,46]. Particularly, this adipokine promotes the recruitment of inflammatory cells to inflammation sites during the early phase of COPD, causing early vascular remodeling, endothelial barrier dysfunction, angiogenesis, and supporting the recruitment of antigen-presenting cells [48]. Moreover, it also alters glucose metabolism by influencing insulin secretion and sensitivity (inducing insulin resistance) and distorts lipid metabolism by increasing the transformation of preadipocytes to mature adipocytes through chemerin-binding receptors. Therefore, chemerin is also involved in adipogenesis as a chemokine, since increased adiposity is thought to be associated with chronic low-grade systemic inflammation [49]. Controlling chemerin signaling may be a promising approach to improve various aspects of COPD-related dysfunction. Therapeutic alterations of chemerin activity could serve as a target for therapeutic approaches aimed at rehabilitating COPD patients through exercise.
  Chemerin can be considered an exacerbation biomarker because it plays a role in pathological processes during acute exacerbation of COPD. Levels of chemerin in the peripheral blood of patients with acute exacerbation were found to be significantly higher than those of healthy controls. With recovery from the exacerbation, the expression level of chemerin decreases [50]. Li C. et al. have demonstrated that the plasma chemerin levels in COPD patients were higher and negatively correlated with blood levels of various lipids. They also found that the circulating levels of both chemerin and lipids are associated with the six-month readmission and mortality rates of COPD patients. This suggests that chemerin might be used as an index for health status assessment and prognosis [51]. Finally, Fang N. et al. observed that salmeterol/fluticasone propionate combined with pursed lip breathing reduced plasma chemerin and lipid levels in COPD patients, consequently reducing the risk of COPD exacerbations and progression [52]. In conclusion, chemerin plays a key role in COPD metabolomics as both an exacerbation and prognostic biomarker.
- *Glutamine*: glutamine has a key regulatory role in antioxidative stress in COPD. Oliveira et al. have reported that the levels of Glutamine are increased in COPD, which may be associated with abnormal skeletal muscle protein metabolism and oxidative stress. However, these data and another report that highlight the increased glutamine, aspartate, arginine, phenylalanine, and branched-chain amino acid levels in patients with COPD are still insufficient to confirm the correlations between amino acid-metabolism and COPD, probably because the amino acids levels depend also on other factors like the BMI and inflammatory reaction than on the presence of COPD itself [53,54].
- *Sphingolipids and glycerophospholipids*: sphingolipids levels, related to altered sphingolipids metabolism, are directly associated with worse lung function outcomes and severity exacerbation. The alteration of glycerophospholipid metabolism was related to airflow obstruction and COPD exacerbations. Thus, sphingolipids and glycerophospholipid represent important exacerbation, and consequently prognostic, biomarkers [55].
- *N,N,N-trimethyl-alanylproline betaine (TMAP)*: TMAP has been associated with a reduced frequency of exacerbations. Although the biological origin of TMAP has not yet been identified and other studies are needed to understand its pathophysiology, it appears to be associated with reduced activity and low oxygen intake in COPD patients.
- *Citrate*: The tricarboxylic cycle metabolite (citrate) is increased and significantly associated with a higher rate of emphysema, which may be related to a dysregulation of the Krebs cycle, with mitochondrial dysfunction and inflammatory–oxidative stress, in smoking patients, which is implicated in the pathology of emphysema. Also, the accumulation of lactosylceramide was identified as a mechanism of emphysema pathogenesis because induces apoptotic–inflammatory responses, aberrant autophagy and the production of reactive oxidation species [56].
- *Glutamylphenylalanine*: in a study conducted on plasma of Chinese COPD patients compared to healthy people, it has been found that glutamylphenylalanine was highly expressed in the healthy group and less expressed in COPD patients with exacerbations. The different plasma levels of glutamylphenylalanine in different COPD stages reflect different proteolytic activities [57].
- *Phenylalanine*: some studies have indicated that phenylalanine, one of the essential amino acids, is related to the severity of COPD. Phenylalanine and other markers such as 3-methylhistidine, acetylated glycoproteins, 3-hydroxypyruvate, serum lipids, and ascorbate could be utilized to categorize COPD patients based on their protein turnover, mitochondrial function, and nutritional status. 3-Methylhistidine, for example, is increased in patients with emphysema due to increased degradation of muscle protein and use of BCAAs (branched-chain amino acids). This suggests an elevated muscle protein turnover in COPD patients with emphysema, which can precede the development of cachexia. The three BCAAs (valine, leucine, and isoleucine) are reduced in COPD patients. This has been interpreted as a result of protein malnutrition and hypermetabolism caused by COPD exacerbation. In cachexia, which is present in 25% of COPD patients, there is altered availability of BCAAs contributing to the anorexic effects of COPD and resulting in low food intake. At the beginning, BCAAs rise with protein degradation. However, after weeks, the blood concentration of BCAAs decreases, and ketone body production increases [57].

## 6. Metabolomics as a Possible Tool in the Differential Diagnosis of Lung Diseases

In the literature, there has been discussion about whether the application of metabolomics can aid in the differential diagnosis of COPD from other obstructive lung diseases and even cancer.

In a study by Maniscalco et al. [58], NMR analysis of exhaled breath condensate (EBC) proved effective in discriminating between COPD and asthma. The study found that levels of ethanol and methanol were significantly higher in COPD patients, while lower levels of formate and acetone/acetoin were observed. The application of metabolomics could help in the differential diagnosis of these two diseases, especially in cases where an overlap between them might be suspected.

Serum and urine metabolites have been tested to differentiate between COPD and OSA using NMR techniques on EBC, but conclusive results have not been obtained [64,65].

De Laurentiis et al. [60] demonstrated how the application of NMR metabolomic analysis can distinguish COPD not only from asthma but also from Pulmonary Langerhans Cell Histiocytosis (PLCH).

Higher levels of 2-propanol and lower levels of isobutyrate were found in COPD when compared to PLCH.

More recently, several studies have begun to analyze the role of metabolomics in the cancer genesis of COPD patients. Indeed, it is unclear how chronic obstructive pulmonary disease (COPD) can affect the prognosis of lung cancer patients, although it is recognized as an independent risk factor for the development of lung cancer. In this sense, attention has been focused on PD-L1 (programmed cell death-1 ligand) [66]. In a study by Polverino et al. [67], PD-L1 expression was evaluated in two cohorts of ever smokers with COPD or NSCLC, as well as in ever and never smoker controls. The authors found that PD-L1 protein expression strongly correlated directly with the FEV1% predicted in all COPD patients. Moreover, Spangenberg et al. [68] found that a metabolite, hydroxyproline, significantly and directly enhances adaptive PD-L1 expression in lung cancer cells. Thus, hydroxyproline could promote the development of an immunosuppressive tumor microenvironment. Therefore, pharmacologically targeting hydroxyproline might aid in controlling the expression of PD-L1 for the treatment of lung tumors.

## 7. Age and Sex as Modulating Factors in Metabolomics of COPD

Ageing can influence metabolomics in COPD patients. Lungs tend to change both functionally and structurally as individuals age. Moreover, it has been evidenced that aging can influence proteostasis, enhance inflammation, and cause mitochondrial stress [69,70]. Changes in methylation of arginine have been reported in older COPD patients [71]. The influence of sex hormones on COPD metabolomics has been investigated. Steroid hormones, such as dehydroepiandrosterone, and their metabolites also exhibit lower levels with age. This phenomenon appears to be more pronounced in COPD and is associated with muscle loss and sarcopenia [72]. Similarly, lower levels of β-hydroxy-β-methylbutyrate were found in patients with sarcopenia and elderly individuals with COPD [73].

Differences in COPD metabolomics have also been reported based on sex. Lower serum carnitine levels were found in females with COPD compared to males with COPD and healthy women [74].

BAL analysis in female smokers with and without COPD revealed differences in lipid metabolites such as octadecenoic, heptadecatrienoic, octadecadienoic, and eicosatetraenoic acids, as well as thromboxane TXB2 [35].

In conclusion, the metabolomic profiles of men and women affected by COPD differ. Specifically, changes in steroids and amino acids are more common in men, whereas abnormalities in lipids and the citric acid cycle are predominantly observed in females.

## 8. Association between Metabolites and COPD Phenotypes

Specific COPD phenotypes are characterized by distinct metabolomic signatures. Changes in carbohydrate metabolites are evident in respiratory secretions of patients with chronic bronchitis. Altered levels of anabolic hormones, proteins, lipids, and other molecules involved in the Krebs cycle have been described in pulmonary emphysema. Differences in the metabolism of proteins and nucleic acids have been observed in COPD patients with frequent exacerbations. Specific alterations in cytokines and eicosanoids metabolism characterize the metabolomic profiles of individuals with asthma-COPD overlap (ACO) and COPD with a high blood eosinophil count [75].

## 9. Conclusions

COPD represents a global health issue, and its biological and metabolic characterization is still a goal to achieve. Metabolomics can now be considered a novel approach for characterizing COPD, transitioning from research settings to clinical applications by analyzing specific metabolites in biomatrices. Although further research is needed and the available research data require validation in larger clinical studies, metabolomics might be able to profile COPD patients in terms of disease severity, exacerbation rate, and treatment response level in a non-invasive and safe setting.

## Figures and Tables

**Table 1:** Comparison between Mass Spectrometry and Nuclear Magnetic Resonance Spectroscopy for Metabolomic Studies.

	Mass Spectrometry	Nuclear Magnetic Resonance Spectroscopy
Detection of Metabolites	Specific chemical classes	All chemical classes
Sample Quantity Needed for Analysis	Few microliters	200–400 microliters
Limit of Detection (Metabolite Dimensions)	Picomolar	Nanomolar
Recovery of the Sample	NO: Destructive technique	YES: Non destructive tecnhique
Time for Data acquisition	10–15 min	10–15 min
Analysis Reproducibility	High (++): targeted approach; Quite low (+−): untargeted approach	Very high (+++)
Metabolites Molecular Identification	Variable (adopted technique)	High

**Table 2:** Levels of Metabolites according to specific Biomatrix in COPD patients. The levels are expressed with + (expressed), ++ (highly expressed), +++ (very highly expressed), ++++ (extremely highly expressed), - (low expressed), -- (very low expressed), --- (extremely low expressed).

Biomatrix	Metabolites (Levels) in COPD Patients
Exhaled Breath Condensate (EBC)	Ethanol and methanol (+++) Lactate, acetate, propionate, serine, proline and tyrosine (++) 2-propanol (+) Formate and acetone/acetoin (--) Isobutyrate (-)
Plasma and Serum	Creatine, glycine, and N,N-dimethylglycine (---)
Bronchoalveolar Lavage Fluid (BALF)	Amino acid (arginine, isoleucine, and serine), fatty acids, and phospholipids (+++)
Urine	1-Methylnicotinamide, creatinine and lactate (--), Acetate, acetoacetate, acetone, carnosine, hydroxyphenylacetate, phenylacetylglycine, pyruvate and a-ketoglutarate (++)
Induced Sputum	Sialic acid, hypoxanthine, xanthine, methylthioadenosine, adenine, and glutathione (++++) Leukotriene E4, D4, prostaglandin D2, E2, 8-iso-prostaglandin E2, F2a, 5-oxo-eicosatetraenoic acid, 12-oxo-eicosatetraenoic acid, and 11-dehydro-thromboxane B2 (++)
Stools	Cotinine, N-acetylcadaverine, N-acetyltaurine (+++)

**Table 3:** Principal Metabolites considered as potential biomarkers in COPD and their biologic activity.

Metabolite	Biomarker Type	Activity and Levels in COPD Patients
Chemerin [45,46,47,48,49,50,51,52]	Exacerbation biomarker; Prognostic biomarker	Chemerin promotes the inflammatory cells recruitment to inflammation sites during the early phase of COPD, causing early vascular remodeling, endothelial barrier dysfunction, angiogenesis and supports the recruitment of antigen-presenting cells; Levels of plasma chemerin are related to mortality rates of COPD patients
Glutamine [53,54]	Disease biomarker; Exacerbation biomarker	Key regulatory role in antioxidative stress; Levels of Glutamine are increased in COPD
Sphingolipids, Glycerophospholipids [55]	Exacerbation biomarker; Prognostic biomarker	Altered sphingolipids metabolism is directly associated with worse lung function and severity exacerbation; alteration of glycerophospholipid metabolism is related to airflow obstruction and COPD exacerbations
N,N,N-trimethyl-alanylproline betaine (TMAP) [56]	Exacerbation biomarker	TMAP levels correlate to low oxygen intake in COPD patients
Citrate [56]	Disease biomarker; Prognostic biomarker	Citrate is increased and significantly associated with a higher rate of emphysema, which may be related to a dysregulation of the Krebs cycle. Dysregulation of the Krebs cycle is a mechanism of emphysema pathogenesis because induces apoptotic-inflammatory responses, aberrant autophagy and the production of reactive oxidation species
Glutamylphenylalanine [57]	Exacerbation biomarker	Different plasma levels of glutamylphenylalanine in different COPD stages reflect different proteolytic activities
Phenylalanine [57]	Disease biomarker; Prognostic biomarker	Phenylalanine is related to the severity of COPD, its reduced levels in COPD patients can be interpreted as a result of protein malnutrition and of hypermetabolism caused by COPD exacerbation.

## Data Availability

Data derived from public domain resources.
